# Adopting standardized cancer patient pathways as a policy at different organizational levels in the Swedish Health System

**DOI:** 10.1186/s12961-023-01073-8

**Published:** 2023-11-27

**Authors:** Petter Fjällström, Anna-Britt Coe, Mikael Lilja, Senada Hajdarevic

**Affiliations:** 1 Department of Nursing, Umeå University, SE 90187 Umeå, Sweden; 2 Department of Sociology, Umeå University, SE 90187 Umeå, Sweden; 3 Department of Public Health and Clinical Medicine, Unit of Research, Education, and Development, Östersund Hospital, Umeå University, SE 90187 Umeå, Sweden; 4 Department of Public Health and Clinical Medicine, Family Medicine, Umeå University, SE 90187 Umeå, Sweden

**Keywords:** Organizational policy, Strategies, Health systems, Organizational levels, Collaboration, Primary health care, Grounded theory method

## Abstract

**Background:**

Standardized cancer patient pathways as a new policy has been adopted in healthcare to improve the quality of cancer care. Within the health systems, actors at different levels manage the adoption of new policies to develop healthcare. The various actors on different levels play an important role and influence the policy adoption process. Thus, knowledge about how these actors use strategies when adopting cancer patient pathways as a policy in the health system becomes central.

**Method:**

The study’s aim was to explore how actors at different organizational levels in the health system adopted cancer patient pathways. Our overarching case was the Swedish health system at the national, regional, and local levels. Constructivist Grounded Theory Method was used to collect and analyze qualitative interviews with persons working in organizations directly involved in adopting cancer patient pathways at each level. Twelve individual and nine group interviews were conducted including 53 participants.

**Results:**

Organizational actors at three different levels used distinct strategies during the adoption of cancer patient pathways: acting as—missionaries, fixers, and doers. Acting as missionaries consisted of preaching the idea of cancer patient pathways and framing it with a common purpose to agree upon. Acting as fixers entailed creating a space to put cancer patient pathways into practice and overcome challenges to this. Acting as doers comprised balancing breadth and speed in healthcare provision with not being involved in the development of cancer patient pathways for the local context. These strategies were not developed in isolation from the other organizational levels but rather, each level interacted with one another.

**Conclusions:**

When adopting new policies, it is important to be aware of the different strategies and actors at various organizational levels in health systems. Even when actors on different levels developed separate strategies, if these contribute to fulfilling the four domains of inter-organizational collaboration, they can work well together to adopt new policies. Our study highlighted that the application of two domains was lacking, which meant that local actors were not sufficiently involved in collaboration, thus constricting the local use and optimization of cancer patient pathways in practice.

## Background

Health systems adopt new policies to achieve diverse goals, including enhancing patient experiences and healthcare professionals work life, reducing costs, and improving population health [1]. Some policy innovations comprise changing managerial practices to utilize resources more efficiently and increase production [2], for example, saving time for healthcare professionals and avoiding instrument waste [3]. Other policy innovations consist of changing organizational processes to improve healthcare provision. In recent years, standardization has gained significant attention [4–6]. According to Timmerman and Epstein [7], standardization is the process of creating uniformity over time and space through agreed-upon rules. Standardized patient pathways within the health system are one example of changes to organizational processes using standardization [8]. Internationally and in Sweden, standardized cancer patient pathways (CPP) have been adopted as a policy to shorten the time to cancer diagnosis and improve the quality of care and health outcomes [9–12]. CPPs consist of pre-determined sets of alarm symptoms associated with different types of cancers and uniform pathways for responding to such symptoms from first suspicion through diagnosis and treatment. Research shows that CPPs contribute to shortened time to diagnosis for some patients [10, 13], but not for patients presenting vague symptoms [14]. Therefore, CPPs as a new policy aiming to improve the process of diagnosis and treatment of cancer provide a unique case to explore how new policies are adopted in the health system and thereby generate new knowledge regarding ways to develop healthcare provision through changing organizational processes.

New health policies, like CPPs, are typically formulated by decision-makers at the highest level of governments, who in turn expect these to be used accordingly by healthcare professionals in practice at the local level [15]. However, actors at different levels of the health system can adopt different strategies when implementing a new policy. The World Health Organization [16] defines health systems as consisting of organizations, people and actions dedicated to promoting, restoring and maintaining health. Health systems comprise a variety of actors, including policy-makers, managers, leaders, healthcare professionals, and patients, who interact at multiple levels [17]. In Sweden, these multiple levels include the national level, which legislates policy and allocates funding, the regional level, which is responsible for administrating healthcare, and the local level, which delivers services to the population through primary healthcare (PHC) units [18]. Ellingsen, Hertzum and Melby [19] highlight that actors on all levels of the health system—national, regional and local—influence policy implementation. Moreover, healthcare professionals at the PHC level play a central role in shaping implementation processes. This may be especially important when a policy such as CPP in Sweden consists of a patient pathway that mostly begins in PHC units. Bardach [20] uses “the game” as a metaphor to turn attention to how players or actors follow rules and utilize strategies during policy implementation to enable or prevent them from being used. The actors’ perspectives are especially important since over time, policies with new rules intended to be used by actors accumulate [15], and a gap may emerge between the policy as written and the policy as performed [21]. In fact, previous research suggests that actors at different levels within the health system purposively act to manage the adoption of new policies [22]. Therefore, an actor-based perspective is needed to better understand how policies are adopted within the health system to improve healthcare provision.

Previous research focuses mostly on actors’ strategies at one organizational level, for example, leader’s use of interventions targeting the management of healthcare provision to reduce unnecessary hospital use among the population [23]. The focus has also been on leadership strategies such as facilitating feedback between leaders and healthcare professionals and prioritizing sustainable interventions with long-term goals in mind to improve the utilization of standardized clinical healthcare provision [24]. Fleming et al. [6] found that two leadership strategies help strengthen organizations competence to implement changes to healthcare provision: selecting leaders suitable for the implementation tasks and coaching healthcare professionals in the intended use. Similarly, Hedsköld et al. [25] shows that leaders play a central role in promoting standardized changes to healthcare provision. Their study found that leaders utilize strategies that value the expertise of healthcare professionals and encourage professional's learning, while acknowledging the need to balance between compliance and questioning of standardized operational procedures in healthcare provision. Moreover, Petersen et al. [26] show that leaders attempt to provide clear job descriptions in line with professionals’ diverse roles, continuous in-service training and workshops to improve effectiveness and responsiveness, while promoting task-sharing across primary- and secondary levels of healthcare (ibid). However, Carlfjord et al. [27] emphasize that these strategies need to target settings with an open organizational climate for the new tool to be used. Additionally, the ways in which a new tool is adopted by different actors in health systems influences how healthcare professionals provide services and to what degree they integrate the new tool into healthcare provision [28].

Meanwhile, other research focuses on the local level, healthcare professionals work to put policies into practice, often with limited resources but with the support of other healthcare professionals [29]. Lipsky [21] points out the important role of individual professionals within welfare organizations, such as health systems, that implement policies by delivering service to citizens. Even though policies are formulated by top-governmental decision-makers, how the healthcare professionals use policies in service delivery influences policymaking at the local level (ibid). CPPs as a new policy to improve time to diagnosis and treatment of cancer in Sweden implies certain changes of the organizational practices of healthcare services not least in PHC units. Nonetheless, research on the adoption of new policies in health systems rarely explores together various actors, from the national to the local level, and their different strategies.

To summarize, current research indicates that putting new policies, such as CPPs, into practice in the health system is not simple. Previously, the focus has been on one organizational level, which overlooks how the actors at different levels of health system develop strategies to adopt new policies. Since various actors on different levels play an important role and influence the policy adoption process, knowledge about these actors' strategies can significantly contribute to improvement of future work with new policies in health systems.

## Method

### Aim

This study aimed to explore how actors at different organizational levels in the health system adopted standardized cancer patient pathways.

### Design

Constructivist Grounded Theory Method (GTM) [30] was used to collect and analyze qualitative interviews with persons directly involved in adopting CPPs within the healthcare organization representing three organizational levels—national, regional, and local—of the health system. Constructivist GTM shares many of the basic tenets of classical GTM, first developed by Glaser and Strauss [31], for example, by focusing the analysis on actions and social processes, using coding, memos and constant comparison, and building theory grounded in data. However, Constructivist GTM, developed by Charmaz [30], recognizes knowledge as socially constructed where the researchers bring their histories, values and ideas to the research and co-create knowledge with research subjects [30]. We considered this method to be the most appropriate to explore the actions and meanings that different actors assigned to the adoption of CPPs at different organizational levels in the health system as a case. Specifically, it allowed us to capture actors’ perspective of the processes of adopting CPPs.

### Case description

The adoption of CPPs in the Swedish health system was selected as our overarching case. This system is publicly funded through taxes for all citizens and provides mostly public healthcare, while including minor private healthcare as well. It has a decentralized structure and is politically governed by both national and regional organizational levels [32]. The national level is responsible for health laws and policies as well as administrating these for the regions. The Swedish parliament determines health policy, including the decision in 2015 to adopt CPPs. The regional level consists of twenty-one regional councils, each with its own administration that governs the provision of healthcare services autonomously through hospitals and PHC units. In our case, we focused on northern Sweden which contains both urban and large sparsely populated areas. At the local level, PHC units act as the first point of contact for patients and provide healthcare services while hospitals provide secondary and tertiary healthcare. As such, PHC units are the main gateway for patients accessing cancer care [33, 34]. In Sweden during 2016, CPPs were designed to be widely used in healthcare including PHC units [12] since 75% of all patients diagnosed with cancer start their pathway in PHC [35]. Therefore, at the local level, we focused on PHC units.

### Sampling the case at different levels of the health system

**Fig. 1:**
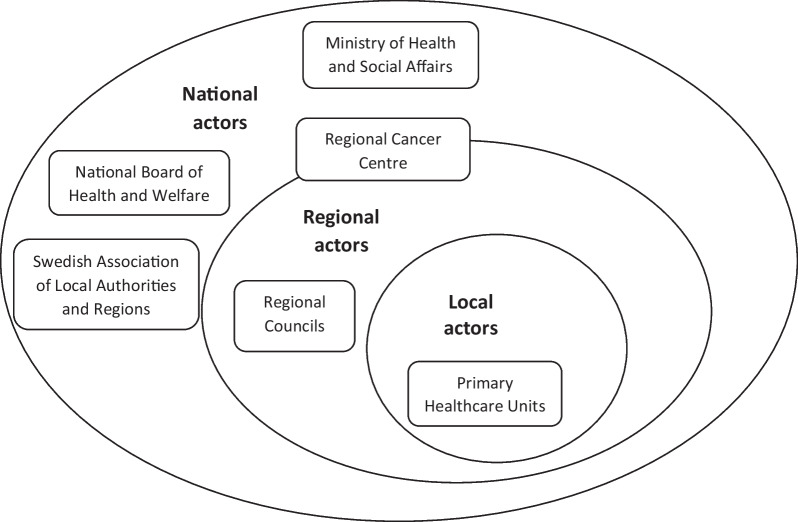
Overview of organizational actors representing three different levels

For our case, we sampled organizational actors directly involved in the adoption of CPPs at each organizational level—national, regional and local—of the health system. See Fig. 1.

For actors representing the national level, we selected four organizations that hold different types of roles concerning the national adoption of CPPs. The first organization was the Ministry of Health and Social Affairs, which is responsible for the country’s social welfare system, including healthcare [36]. Secondly, we selected the National Board of Health and Welfare, responsible for ensuring the quality of healthcare to the population, evaluating healthcare and developing national guidelines for healthcare services to follow [37]. They manage, for example, the classification of care measures containing codes for healthcare interventions, which are used to measure the time intervals of CPPs. Thirdly, we selected the Swedish Association of Local Authorities and Regions, an organization that represents and advocates for the country’s regions and municipalities, in the national policy arena [38]. In the case of CPPs, their responsibility in the health system is to sign collective agreements between the national government and the regional councils. Lastly, we selected the Regional Cancer Centre in northern Sweden which collaborates on a national level to create more equal cancer care nationally [39]. At the national level, their role is to advise and perform tasks for the three previously mentioned organizations regarding cancer care and act as a link between the national level and regional councils.

For actors representing the regional level, we selected three organizations representing two different types of roles for the adoption of CPPs at that level. The Regional Cancer Centre in northern Sweden links regional councils together to coordinate cancer care. They provide support to the regional councils in the adoption of CPPs and other new ways of working in cancer care. Furthermore, healthcare in northern Sweden is governed by four regional councils that cover half of the country's landscape with approximately 10% of inhabitants and constitutes both large sparsely populated areas and some cities. We selected two regional councils from northern Sweden, Västerbotten and Jämtland Härjedalen, which have 10,000 and 4000 employees respectively. These are the largest and smallest regional councils in terms of organizational size in northern Sweden. In 2021, Region Västerbotten had 38 PHC units and three hospitals available for 274,563 residents within an area of 54,664 km^2^ while Region Jämtland Härjedalen had 26 PHC units and one hospital available for 132,054 residents within an area of 48,935 km^2^.

For actors representing the local level, we selected six PHC units in the regional council of Västerbotten. At the local level, healthcare services are provided within regions through a specialized organization that embodies a variety of licensed healthcare professionals who are relatively autonomous in their professional practice and work in multiple specialized healthcare units. Each PHC unit held similar types of roles for the adoption of CPPs at this level but attended catchment populations of different sizes (small, medium, and large) and concentrations (urban-sparsely populated areas). We selected PHC units with a variation in unit size and location to reflect the health system in northern Sweden at the local level.

### Recruitment of participants and data collection

To recruit participants, we contacted persons working in the selected organizations identified as being directly involved in the adoption of CPPs. An overview of the actors representing the different organizations and levels in our study and the number and type of interviews conducted with each one is presented in Table 1. Individual interviews were conducted with persons at the national level because they held singular positions within their respective organizations as policy experts. Also, individual interviews were performed at the regional and local levels with managers and leaders that required their direct involvement in developing the new CPP policy or spreading the new policy to the local level. In contrast, at the local level, the entire PHC units were expected to adopt the new policy in practice, make it more sensible to conduct interviews with groups of healthcare professionals, to capture this collective experience. Twelve individual interviews were conducted with participants involved in the adoption of CPPs, who held leadership positions such as advisor, coordinator, manager, project leader, or director. Nine group interviews were conducted across six PHC units with healthcare professionals (21 PHC nurses and 20 PHC physicians). A total of 53 participants (75% women) were included in the study.

**Table 1:** Overview of organizations, organizational levels, and collected data

Organizations	Organizational level	Collected data
Ministry of Health and Social Affairs	National actor	1 individual interview
The National Board of Health and Welfare	National actor	2 individual interviews
Swedish Association of Local Authorities and Regions	National actor	3 individual interviews
Regional Cancer Centre	National actor and regional actor	1 individual interview
Regional Councils	Regional actor	4 individual interviews
Primary Healthcare Units	Local actor	1 individual interview 9 group interviews

When participants were recruited, we gave them information on the study and invited them to participate. All invited actors from each level accepted to participate. Two of the authors conducted the interviews, one with clinical experience as a nurse (SH) and one with policy experience as a sociologist (ABC), thereby complementing one another with insider and outsider perspectives of the health systems during the data collection process.

All participants gave their voluntary and informed consent before the interview. The interviews were arranged at their workplace or by telephone according to the participants’ requests. We used a semi-structured interview guide with open-ended questions that asked participants about the process of adopting CPPs and how the participants as actors at their given organizational level were involved in the process. Follow-up questions and additional questions were developed to probe the specific experience of actors at each level and emerging categories during the data collection. In total, data were collected through twelve individual and nine group interviews between May 2018 and November 2019. The interviews lasted from 18 to 70 min (mean 42), were audio recorded as well as documented in notes during the interviews, verbatim transcribed, and further inserted into MAXQDA 2018, a software data program for coding and analyzing qualitative data.

### Data analysis

During data analysis, we followed the coding techniques of the Grounded Theory method to perform a constant comparison between empirical data, codes, and memos [30]. We started with the initial coding phase of the individual interviews and wrote memos to involve ourselves with the data. We coded transcribed interviews line-by-line, interacted with each fragment of data, and used active codes to focus on actions and meanings. After the initial coding of the entire data material, the focused coding phase was carried out where the initial codes were sorted and grouped into categories. We continued to go back and forth between these coding phases using constant comparison to analyze and theorize the codes into categories and the relationships between them. In the theoretical coding phase, we interpreted our categories as ways of acting within the different levels of the health system and developed our theoretical codes. In the analysis, we in the research group handled our preconceptions by discussing our findings together during each phase and maintaining an open mind grounded in the data. At the same time, we used memos to stimulate ideas during the analysis and identify patterns in the data. We started by exploring the multiple interactions between different organizations at different levels during the adoption of CPPs. This analysis led us to construct three patterns of action presented in the results section. The first author (PF) performed all the coding techniques while all the other authors have been involved during each analysis phase and provided varied perspectives to elevate the analysis and develop the theoretical modeling from the actions.

## Results

Our analysis captured how actors at each of the three different organizational levels in the health system adopted CPPs by developing respective actions. The three ways of acting were: acting as missionaries, acting as fixers, and acting as doers, see Table 2. Drawing upon the Theoretical Code Family of strategy [40], we theorize these actions as conscious strategies developed by actors at each organizational level to handle the adoption of CPPs. The different strategies used to adopt CPPs at the national, regional, and local levels suggest that actors at each level held distinct functions, faced distinct conditions, and had distinct possibilities to act. Despite these different ways of acting, actors did not develop their strategies in isolation from those at the other levels. Instead, they accounted for the other levels by interacting with one another. Next, we demonstrate in-depth the three ways of acting.

**Table 2:** Overview of categories and properties

Properties	Categories
Preaching the idea of CPPs as a way of working	Acting as missionaries
Framing CPPs with a common purpose	
Creating space for CPPs to fit into healthcare	Acting as fixers
Managing constant challenges	
Balancing breadth and speed in PHC provision with CPPs	Acting as doers
Producing PHC services daily as usual	

*CPP* cancer patient pathways

*PHC* primary healthcare

### Acting as missionaries

Acting as missionaries consisted of two properties that encompassed preaching the idea of CPPs all over the country and framing CPPs with a common purpose. The national actors were the ones acting as missionaries. The first property meant preaching CPPs as a necessary solution to achieve efficient and equal healthcare and packaging it as something vital for the health system to use in practice. National actors believed that CPPs were needed to make patient trajectories more efficient while at the same time help ensure equal healthcare nationally. National actors described feeling obligated to address problems of inefficiency in the health system caused by bottlenecks that prolonged the time to diagnosis and treatment for patients. They saw CPPs as a solution to this, as captured by the following quote by a participant from the national level:

“I think that CPP puts the finger on certain organizational solutions that are better or worse from a CPP perspective and may be better or worse from the patient perspective to get this patient pathway uh… So, it probably clarifies where you have them… like the bottlenecks… and what other problems there are” (Individual interview 4).

As this quote illustrates, national actors saw CPPs as pinpointing inefficiencies in the organization that halted the patient care trajectories and would resolve these problems. They were convinced that CPPs would facilitate and speed up patients’ care trajectory through the health system. At the same time, CPPs were considered solutions to getting all actors in the health systems to put patients’ needs first, as described by another participant from the national level in the following quote:

“It is possible to set up a good logical patient pathway for examining patients with suspected disease, I am completely convinced of that. But then everyone must also want to focus on it, that is, we focus on the patient’s process all the time when setting up the healthcare structure. It is not other interests or forces that control how the process is set up. It is the patient’s journey that is central” (Individual interview 3).

As this quote describes, a belief existed that CPPs would help ensure that only patients’ health needs shaped their care trajectory. Therefore, national actors preached that the standardization offered by CPPs would have positive effects and they emphasized these effects in their communication with regional actors. As our interview data shows, national actors perceived strong political agreement among them, which they considered as crucial to adopting CPPs, as described by a participant from the national level in the following quote:

“It (CPP) has had a good impact because it has such national reach and political unity, both the opposition and the current government believed in it. Cancer care, with all the achievements in the regions, should declare (success) ... Cancer care has succeeded in so much now, and it is time for others to also learn from cancer care” (Individual interview 2).

Thus, national actors not only believed in the idea of CPPs in theory but were also believers in its positive effects on cancer care. They believed in the CPPs as a success in cancer care and were confident that the CPP way of working could improve healthcare generally in the health system as well. This facilitated their action of preaching CPPs as a cure-all for all kinds of problems existing in healthcare.

The second property of acting as missionaries meant framing CPPs as a common purpose around which to unify the twenty-one autonomous regional councils. This framing was intended not only to convince regional actors to adopt CPPs but also to address fragmentation in the health system. National actors used CPPs to pull together the invisible but existing distance among the different regional councils and between regional councils and PHC units. To do this, they proposed CPPs as an intervention with goals of ten to twenty years in the future in mind to cooperate around a way of working and make CPPs last longer within the health system. A participant from the national level described it like this:

“Big initiatives for citizens, big policy campaigns, they are not that sustainable, about a year or something like that. We also know that it takes a lot of effort to work with it. That's why initiatives like this (CPP), such a reform and an investment, may have more significance because it is more sustainable since the knowledge is in people's heads now” (Individual interview 2).

As such, national actors framed CPPs such that healthcare actors could collaborate around a common purpose and thereby adopt it in all regions. It was a strategy to make CPPs have a lasting impact as a new way of working in the health system and engrave CPPs in healthcare practice. Additionally, national actors created and used administrative and funding strategies to ensure that CPPs was adopted throughout the health system. In interview accounts, national actors depicted monitoring time intervals to diagnosis and required regular reporting from the regional councils that measured the effects of CPPs; they employed rhetorical techniques and offered targeted funding to convince and motivate regional councils to adopt it. They described how they monitored the time intervals through a coding system of the classification of care measures that the healthcare providers already used. They also mentioned distributing national funds to reward the regional councils when CPPs was adopted and used in healthcare practice.

Meanwhile, the national actors respected their boundaries of not deciding in spaces where they do not belong. As a respondent from the national level expressed: *“We do not want to involve ourselves too much from the national level. We don't want to go into too much detail and govern in that way”* (Individual interview 5). Still, the national actors tried to convince regions to use CPPs adapted to their prerequisites. For example, a participant from the national level described how they invited the top management from the regional councils to recurring meetings to convince them to use CPPs:

“We here (nationally) are convinced of its (CPP) effect. We can convince maybe three, four or ten of the regional directors that we gather here, or they gather, and we go there, and try to convince them that now you can go home and do this and then ten of them do it but not twenty-one, therefore not everyone has been convinced. Then you must invite them again and that type of meeting, in such a chain, must be managed” (Individual interview 3).

As this quote shows, national actors could not authorize an order to use CPPs and instead actively attempted to convince the regional actors to make use of CPPs in healthcare services.

### Acting as fixers

Acting as fixers had two properties that encompassed creating a space for regional councils to incorporate CPPs and overcoming potential challenges. The regional actors were the ones acting as fixers. The first property entailed creating space to fit CPPs into the organizational structure of healthcare, by among other things, managing the conditions needed to use them. Regional actors strategized to shape the conditions for local actors to take on working with CPPs and handle the adoption of multiple CPPs. Acting as fixers, they sought to provide stability and predictability while at the same time make room for the expansion of different CPPs according to the type of cancer, which were being developed at the national level each year. As respondents described, they developed ways to manage CPPs between the different parts of the organization that were involved in adopting CPPs to ensure that it would be put in place across healthcare services. The regional actors worked to adapt CPPs to their local contextual conditions and act accordingly. For example, most regions adopted a new coordinator to administrate CPPs regionally, yet the coordinator had a different function depending on the size of the region and the organizational structure itself as described by a participant from the regional level in the following quote:

“We have adopted it (CPP coordinators) in a slightly different way than what has been done elsewhere, some regional councils with few hospitals have done it on a region-wide level, the coordination centre in XX (regional council) has such a thing. We will also continue to sharpen our organization with coordinators, but we have networks with them that we keep together. The coordinators in those networks are very important because they notice when something goes wrong in the health system, and they oversee those who monitor the referral flow” (Individual interview 8).

As this quote also explains, the regional actors valued and used coordinators to manage the CPPs within the health system which helped improve the flow between of patients referred to CPPs, for example, between primary and secondary healthcare. Regional actors strived to connect CPPs with the already existing organizational structure in their regional health system and operationalized CPP as a way of working in healthcare practice. From the interviews, the standardized dimension of CPP was viewed as leading to a more efficient utilization of limited resources, both time and personnel, as described by a participant from the regional level: *“The development of cancer care (with CPP) has shown that it is possible to do really good things and do it much faster than we first thought, and with that, we use resources better”* (Individual interview 6). As this quote illustrates, regional actors were motivated to operationalize CPP as a way of working in the health system and saw the possibility to increase the efficiency in the provision of healthcare for other diseases in the health system as well. Additionally, acting as fixers also included linking different regions with one another and thereby spreading ways to adopt CPPs between regions. It was primarily the Regional Cancer Centre, created before the adoption of CPPs, that acted as link between different regional councils, as the following quote from a participant at the regional level portrays:

“We have a collaboration group between regional councils and a steering group where the healthcare directors of the various regional councils are included. They were very much in agreement that it was clear that we had to continue working together. Because there are still many things that do not work perfectly (in CPPs). We need to talk to each other and exchange experiences so the regional cancer centres will continue to link the regional councils through a collaboration group” (Individual interview 7).

At the regional level, the Regional Cancer Centre took it upon themselves to facilitate collaborations between regional councils and together create a space for CPPs within the healthcare structure. The regional cancer centre linked the separated regional councils together since the regional actors needed arenas to learn from each other and thereby improve ways of working with CPPs.

The second property of acting as fixers encompassed overcoming the challenges to CPPs. Regional actors were preoccupied with overcoming challenges and finding solutions to them. Factors such as limited resources—financial, time, and personnel—imprinted the health system. In the early phases of CPPs, regional actors awakened and realized how unprepared they were when the first five CPPs were introduced to the regional councils. In the interview accounts, they realized that organizational structure of healthcare was fragmented, that bringing in CPPs to isolated PHC units demanded a lot of work, and little work had been done to prepare for the coordination that was needed between healthcare levels for CPPs. The regional actors described CPP as a top-down initiative where the planning phase in regions was practically non-existent, and they had to fix their organization on the go. A participant from the regional level described challenges in the early phases of adopting CPPs in the following quote:

“We started too late, and it was very stressful and extremely difficult from the beginning. So, if we had an additional year before, then you would have had time to get information out many times before it (CPP) would start, that alone would have been incredibly valuable. That was the big obstacle from the beginning and the challenge that we had with us for quite some time. To get people on board and to build the rails while the train (CPP) is coming from behind. It has been hard, I must say” (Individual interview 9).

As this quote illustrates, regional actors managed constant challenges to keep up with CPP's rapid introduction early on since it was a top-down approach to impose a new way of working in healthcare in a short time. Regional actors were responsible for constructing the conditions for local adoption of CPPs and motivating their use in healthcare. Yet, they did not have the time to resolve unanticipated issues along the way. In the interviews, they acknowledged that the PHC units were separated from hospitals. The solution was to include a few local representatives from PHC units in meetings or to engage a small number of individuals as champions, mostly physicians, that could personally encourage the use of CPPs in PHC units. Still, they directly focused on CPPs as a solution to cooperate and gather around a new way of working to improve healthcare practices as described by a participant from the regional level: *“CPPs influence that primary and secondary healthcare had to sit down and look at what their common processes look like”* (Individual interview 10). The regional actors used CPPs as the general solution and jumped directly to what they could do to overcome the challenges of adopting CPPs in the regional organizational structure of healthcare, neglecting other possible factors to the problem. As respondents described, they learned to manage the constant challenges through collaboration between different levels and departments. One participant from the regional level emphasized the importance of collaboration as described in this quote: *“It has become a collaboration, with primary healthcare, radiology, pathology, and with everyone involved. They have been involved in every initiation of a new CPP”* (Individual interview 9). Regional actors had to handle the fit between CPPs and different aspects of the organizational structure of healthcare. However, they let local actors in PHC units solve the challenges of managing CPPs in daily healthcare services with limited resources. Instead, regional actors’ strategy involved using CPPs as the general solution to increase efficiency in healthcare and stimulate collaborations. The challenges in the organization were something that they thought they could fix with CPPs and therefore overcome.

### Acting as doers

Acting as doers had two properties that encompassed balancing breadth in PHC with faster patient trajectories when using CPPs and producing healthcare services to patients as usual when they lacked the possibilities to optimize CPPs. Local actors, in the form of PHC units, were the ones acting as doers. The first property comprised balancing speed and breadth in healthcare provision by using CPPs to complement existing practices of managing patients and facilitating patient flow. Local actors prioritized assessing alarm symptoms and identifying possible serious diseases, such as cancer, and CPPs fitted well in this work. On one hand, they took on CPPs as a tool to diagnose cancer fast, and on the other, they maintained their function as the first contact and a filter for patients with multiple diseases. According to participants' accounts, they strategized to maintain an open mind to CPPs and be willing to learn about them. A participant from a PHC unit described that CPPs applied to them at the PHC unit like this: *“With CPPs, the path is clear how we should act. The standardized and uniform way makes it easier to use and increases equality for patients”* (Individual interview 11). Local actors easily used CPPs in healthcare provision because it fitted well with their practices of managing patients. Participants depicted being alert to possibility that patients may have cancer. During a group interview at a PHC unit, a nurse described their role as first contact when suspecting cancer like this: *“If I talk to the patient on the phone and I assess that you have alarming symptoms, then I schedule an appointment with the physician immediately”* (Group interview 5). Thus, local actors had confidence as practitioners to provide an entrance into healthcare for multiple diseases when they suspected a disease.

Furthermore, local actors worked with CPPs to provide faster access to cancer care for patients while they used their professional expertise to filter and assess each unique patient. According to the interviews, local actors were accustomed to filtering patients with a lot of different symptoms and doing whatever they could to provide the best possible healthcare to patients. They mostly concentrated on alarm symptoms (i.e., red flags) to decide whether a patient should be included in CPPs or not as described by a participant at a PHC unit in the following quote:

“We are the patient's first contact many times... or most times... and we identify these symptoms that could be cancer… yes... and then refer it on. Because the role of primary healthcare is a lot of sifting and looking at what we can do or not... these things (in CPP), these flags, the red flags, they are very important for us to discover” (Individual interview 11).

The local actors portrayed balancing their breadth by functioning as a filter for patients’ first contact while focusing on alarm symptoms to enable fast access to cancer care when they suspected cancer. Even though local actors did not attempt to resist CPPs, they strategized to protect their professional expertise upon which to base whether to include a patient in a CPP. Furthermore, the local actors acted as the experts in the context of PHC and drew upon their clinical judgment to dispute CPPs or, when it was relevant, to utilize it. Our interview data show that they made exceptions from CPPs criteria if their clinical assessment pointed them in other directions than cancer.

The second property of acting as doers encompassed producing healthcare for patients as usual because they were not involved in developing CPPs for patient encounters in PHC. Local actors described not being involved in the development of CPPs, and therefore felt they lacked sufficient knowledge about CPPs to completely abandon their usual way of working. In the interview accounts, they depicted having received information on CPPs from regional-level meetings. However, only some participants attended these meetings and very few were involved in discussions about the development of CPPs at the regional level or got to learn about CPPs from educational initiatives. For example, a physician at a PHC unit described missing out on CPP information like this: *“We have received information (about CPP) from a few different sources. However, I have searched for information on the internet and ended up on some websites and reacted with… Oops…. look so many CPPs there were now suddenly”* (Group interview 1). Another physician in the same group was disappointed in the top management provision of CPP information at the local level as described in the following quote: *“Physicians here at the primary healthcare unit have given us sufficient information about CPPs but I can't say that it has been communicated so clearly from the top management”* (Group interview 1). The local actors portrayed being excluded from the development of CPPs and had almost given up on influencing the ways CPPs could be optimized to be better compatible with their routine work. Since the local actors were not involved in the development of CPPs, their adoption of CPPs was gradual and performed while carrying out their usual ways of working by identifying alarm symptoms of possible cancer. Our interview data shows that the adoption of CPPs did not have a clear starting date in PHC units. A physician from a PHC unit describes it in the following quote: *“At the PHC unit, we have not adopted CPPs in one fell swoop. Instead, it is something that has been starting to be used gradually”* (Group interview 2). Local actors gradually changed ways of working as time passed and CPPs did not seem to reach the local level as a tool to improve the provision of healthcare as intended by the national actors. Meanwhile, in our interviews, participants could not only focus on the ways of working with CPPs and drop everything else as described by a physician from a PHC unit in the following quote:

“Most cancers are detected in primary healthcare, but at the same time, it is only a small part of our patient flow. We manage so many patient pathways, so it (CPP) becomes part of everything else that we must manage. We deal with so many different things such as mental illness and mild psychiatry as well as all other metabolic diseases and so on” (Group interview 2).

As this quote illustrates, local actors provided healthcare as usual and managed patient pathways for multiple diseases and CPPs were one of these. Local actors felt a strong sense of belonging in their autonomy as professionals and were proud to be able to handle their duty to take care of their patients within their community. To some extent, local actors were not left out only because the regional top management neglected them. They also isolated themselves and chose to control their work in the PHC unit while mainly focusing on their responsibility as healthcare professionals.

## Discussion

Our study found that organizational actors at three different levels of the health system used distinct strategies during the adoption of CPPs: acting as—missionaries, fixers, and doers. These strategies were not developed in isolation from the other organizational levels but rather as each level interacted with one another following the common goal to adopt CPPs while having different perspectives on it. We draw upon the literature on inter-organizational collaboration (IOC) to shed light on our findings. Himmelman [41] defines collaboration as exchanging information, altering activities, sharing resources, and enhancing the capacity of another for mutual benefit and to achieve a common purpose. Actors in IOC typically use different strategies to achieve common goals [41]. This helps explain our findings on the adoption of CPPs in a health system where several professions at different organizational levels work. Our results suggests that actors shared the goal of aiming to improve health outcomes among patients yet diverged in their strategies along each level of the health system. Moreover, our findings demonstrate that actors from the different levels exchanged information, carried out activities to alter ways of working, and shared resources in the adoption of CPPs to achieve this common goal. However, they experienced that the efforts to strengthen capacity at all levels, integrate their different perspectives, and unify the policy adoption the policy for mutual benefits were limited. Furthermore, our study highlights that actors might focus on their own specific level without the ability to create a shared understanding of the adoption process of CPPs by involving actors from all levels of health system. Even though the organizational levels in some way interacted with each other, the same degree of investment was not made in aligning all organizational levels of the health system to work towards the new common goal and CPPs became imposed from the top-down instead.

Following Longoria [42], IOC consists of four domains. Our results indicate that IOC was achieved on two of these domains, which enabled actors on different organization levels to work together in the adoption of CPPs. The first domain presented by Longoria [42] is simply that IOC is a joint activity in the form of a relation between two or more organizations. In our study, the actors on all three organizational levels had a pre-established relationship with one another and pursued their respective strategies to adopt CPPs in the health system. For example, national actors provided information regarding CPPs but also worked to convince the regional and local levels to adopt CPPs in their organizations. Other studies have found that actors from different healthcare organizations use similar strategies and work together in IOC to adopt new policies and re-organize work aiming to improve healthcare through sharing knowledge and information [43, 44]. Longoria’s [42] second domain of IOC is that structural properties emerge from the relationship between organizations, i.e. the relationship exists within an organizational structure which links a collective body of organizations and actors together. In our results, new structures and roles were created to facilitate CPPs in healthcare provision, such as regional networks or centers, and CPP coordinators. Especially by acting as fixers, the regional actors focused on creating these new structures. Moreover, collaborative groups between regional councils were created that enabled sharing and exchange of information to facilitate the adoption of CPPs nationally. Similarly, Kousgaard et al. [45] show structural changes and the creation of roles intended to link PHC and social structures to improve IOC in healthcare service delivery where PHC physicians were allocated time to focus on the task of collaborating with other involved organizations. Also, von Heimburg and Hakkebo [46] found that establishing a new strategic unit implied reinforced structure and developing leadership roles and networks which facilitated the adoption of new policies. In our case, achieving the first two domains in Longoria’s model facilitated the adoption of CPPs as a new policy in practice.

In contrast, IOC was not achieved regarding two of Longoria’s domains focusing on the intentional planning process and synergistic development, which constricted actors on different organization levels from working together in the adoption of CPPs. The third domain according to Longoria focuses on IOC as an intentional planning process with mutually shared organizational goals [42]. Our results indicate that the planning process of adoption was mainly developed from the national actors’ perspective rather than the regional or local actors’ perspective. Yet, national and regional actors interacted relatively well because of shared goals of increased efficiency and reduce inequal healthcare. Meanwhile, these levels interacted inadequately with the local PHC level. Our results show that the local actors worked as best they could to produce healthcare services as usual in their adoption of CPPs, but they were barely involved in the planning process itself. This suggests that different perspectives of organizational levels did not meet which was a barrier to work together in the adoption of CPPs. Similarly, previous research shows that barriers on different levels such as lacking coordination and leadership as well as incompatible organizational structures, missing important actors and uncertainty could impede the IOC and influence the outcome of the integration of new healthcare policies [47]. The fourth domain of IOC according to Longoria consists of synergistic development which is the process of organizational actors’ collaborating to accomplish something greater together than by themselves [42]. However, our results indicate that each organizational actor was pursuing their strategy rather than working to accomplish something greater together. Moreover, that local actors, the PHC units, were barely involved limited the synergistic development because their engagement in the adoption of CPPs was not optimized. Even though IOC is often used as means to generate intended synergistic results, this is not always the case in organizations, since the policy intentions and the capacity to accomplish them do not always fit [42]. Low et al. [48] found that local actors from PHC level perceived a dilemma when top-management mandated immediate action according to a new policy that affected already ongoing initiatives in combination with insufficient resources issues. In our case, not achieving the last two domains according to Longoria hindered the adoption of CPPs as a new policy in practice.

To summarize, in our result, the various strategies used by the different actors, the absence of coordination between them and the lack of recognition of local actors by national and regional actors constricted the ability of the different organizational levels to work together in a unified way to adopt CPPs. Adding to the complexity, the decentralized structure in the Swedish health system reinforces the division of task-sharing and responsibility between involved actors at different organizational levels. Thus, the involvement of actors from different levels in policy adoption and their perceived togetherness through planning common goals becomes central. Our findings indicate not only that each level had different functions and possibilities to act accordingly, but also that the different perspectives on the task may have influenced their relations, interests in collaboration and engagement in the adoption CPPs. Gray and Purdy [49] describe power as an actor-based activity intended to influence and ‘power to’ as the capacity to dispose of own actions. In our results, we observed that all three levels had the power to dispose of actions within their organizational level. However, especially the national and to some extent the regional level had greater power to decide over the actions of the local PHC actors. Our participants described that the need to consult and involve the local PHC actors during the early work phase of adoption of CPPs was not recognized by the actors from national and even regional levels, and some PHC professionals like nurses were not involved when providing information on CPPs to the local actors. Meanwhile, previous research has shown that involving healthcare professionals early in the adoption process is important to promote their readiness for change and continual support after the change [50, 51]. In our results, the national actors presented CPPs as a general solution to unify patient pathway within the health system and the regional actors conformed it and let the local actors handle the compatibility between CPPs and local practices themselves. Unexpectedly, it was the local PHC actor’s responsibility to handle the adoption of CPPs in healthcare provision with limited guidance from regional actors, and the national actors avoided involvement in local affairs. Moreover, research shows that barriers to new policies being adopted in healthcare practice can be caused by healthcare professionals’ lack of understanding regarding possible benefits and their negative attitudes and behaviours towards the new policy. Brooke-Sumner et al. [52] highlight that these barriers are often influenced by health system constraints such as high workload related to limited resources and frequent policy changes. Involving local actors in policy adoption has been found to strengthen healthcare professionals’ capacity, beliefs, and confidence in its use [24]. Involving actors from different levels and working together in early phases is needed to improve the capacity for IOC during the adoption of new policies in practice. This could facilitate and improve the process of the adoption of CPPs in northern Sweden.

### Strengths and limitations of the study

This study benefited from using GTM because it allowed us to explore our case in an open, flexible, and systematic way. The multidisciplinary research team (nursing, social and medical sciences with a gender-equal group) brought together expertise from PHC, cancer, and policy research to contribute different perspectives during data collection and analysis. The group’s different perspectives and backgrounds allowed us to handle actively our preconceptions in data collection and analysis, and to ground emerging categories in participants’ actions and meanings. Limitations were that a policy analysis or observations of CPPs use in practice might have reinforced our study design. However, we chose to focus on the involved actors’ perspectives and actions by interviewing 53 participants from different levels of healthcare to capture the adoption process of CPPs as a new policy in the health system. Another limitation was that we restricted the regional and local setting to northern Sweden. Yet, we included two regional councils of varied sizes and a variety of bigger and smaller PHC units to be able to reflect upon the meanings of the organization of healthcare services related to the adoption of CPPs in both urban and sparsely populated areas.

## Conclusion

Our study indicates the significance of collaboration and common goals among different actors during the process of adopting new policies within the health system. The use of different strategies among actors at the various levels of the health system appears to be necessary. Nonetheless, involving all actors and caring for each other’s perspectives influences the policy adoption process, including the match between intentions and practice. Even when actors on different organizational levels developed different strategies, if these are fulfilling the four domains of IOC, the process of adopting new policies can work well. Furthermore, the involvement of actors from all levels of the health system in developing common goals can facilitate and optimize the adoption of new policies at all levels. Thus, actors from all levels within the health system from local PHC actors to actors on the national level must be involved and supported to strengthen their local capacities to work together for a new policy, such as CPP, to be used practically and improve the quality of care for patients.

## Data Availability

The datasets generated and/or analyzed during the current study are not publicly available due to the data including information that could compromise research participants’ privacy and consent but are available from the corresponding author on reasonable request.

## Abbreviations

CPP: Cancer patient pathways
IOC: Inter-organizational collaboration
PHC: Primary health care
